# Optimism and work–life balance in relation to infertility self-efficacy in IVF couples: a dyadic approach

**DOI:** 10.3389/fpsyg.2026.1819261

**Published:** 2026-09-30

**Authors:** Keren Turgeman-Lupo, Ya’arit Bokek-Cohen, Amir Wiser, Einat Haikin Herzberger, Netanella Miller

**Affiliations:** 1 School of Management & Economics, The Academic College of Tel Aviv-Yaffo, Tel Aviv-Yafo, Israel; 2 School of Behavioral Sciences, Netanya Academic College, Netanya, Israel; 3 Meir Medical Center, Kfar Saba, Israel; 4 Faculty of Medicine, Tel Aviv University, Tel Aviv, Israel; 5 OB/GYN Department, Maayanei Hayeshua Medical Center, Bnei Brak, Israel

**Keywords:** dyadic approach, *in vitro* fertilization, infertility self-efficacy, personal optimism, work–life balance

## Abstract

**Introduction:**

Infertility treatment is a prolonged medical and psychosocial stressor, and infertility self-efficacy reflects individuals’ perceived capacity to cope with treatment demands. Research has rarely examined how personal and work–nonwork resources are jointly associated with infertility-specific coping beliefs within couples. Drawing on Conservation of Resources theory, this study examined personal optimism (PO), perceived work–life balance (WLB), and infertility self-efficacy (ISE) among women undergoing *in vitro* fertilization (IVF) and their male partners, including an indirect association from PO to ISE through WLB and its possible difference between partners.

**Methods:**

The analysis included 44 employed heterosexual couples (*N* = 88) undergoing IVF. Both partners independently completed measures of PO, WLB, and ISE. Partner-specific indirect-effect models were estimated separately for women and men, adjusting for the other partner’s corresponding scores. Between-partner contrasts in the PO–WLB and indirect associations were tested in a simultaneous distinguishable-dyad path model using couple-level bootstrap resampling. Given the cross-sectional design, indirect effects were interpreted as statistical associations rather than evidence of causal mediation.

**Results:**

Among women, PO and WLB were each positively associated with ISE. However, PO was not significantly associated with WLB, and the indirect association between PO and ISE through WLB was not statistically significant. Among men, PO was positively associated with WLB, and both PO and WLB were positively associated with ISE. In addition, a significant positive indirect association between PO and ISE through WLB was observed among men. A direct dyadic contrast showed a stronger PO–WLB association among men than among women, although the confidence interval narrowly excluded zero. However, the direct comparison of the partner-specific indirect associations did not indicate a statistically significant difference between women and men.

**Conclusion:**

PO and WLB were independently associated with ISE in both partners. Among men, but not women, a significant indirect association between PO and ISE through WLB was observed, although the direct comparison of the partner-specific indirect associations was not statistically significant. These findings suggest that psychosocial care during IVF may benefit from attention to ISE, PO, and WLB. Longitudinal and intervention research is needed before clinical recommendations can be drawn.

## Introduction

Infertility is a prevalent global health condition with profound psychological and social implications. The World Health Organization estimates that approximately one in six individuals worldwide experiences infertility during the reproductive years (World Health Organization, 2023). The use of assisted reproductive technologies (ART), including *in vitro* fertilization (IVF), has expanded steadily, and many couples face prolonged or repeated treatment cycles. Beyond its biomedical demands, IVF commonly entails anxiety, depressive symptoms, relational strain, and substantial psychological distress (Ying et al., 2016). Psychological resilience is positively associated with fertility-related quality of life, with self-efficacy and coping strategies identified as important mechanisms in this association among women receiving infertility treatment (Xu et al., 2025). Identifying resources associated with perceived coping capacity during IVF is therefore an important clinical and public health priority.

Infertility self-efficacy (ISE) refers to individuals’ perceived ability to cope with infertility and its treatment-related demands (Cousineau et al., 2006). It is associated with emotional regulation and adaptive coping among individuals undergoing infertility treatment (Cousineau et al., 2006; Ersen et al., 2023). Higher ISE has also been linked to lower depression, anxiety, and stress (Cousineau et al., 2006; Mirzaasgari et al., 2022), although Mirzaasgari et al. (2022) found no statistically significant association between ISE and pregnancy outcomes following assisted reproductive treatment. Intervention studies indicate that ISE can be strengthened during infertility treatment (Altiparmak and Derya, 2018; Yanık and Kavak Budak, 2024). Taken together, these findings establish the clinical relevance of ISE as a coping belief. However, less is known about how broad personal resources become connected with this infertility-specific sense of capability, particularly through contextual conditions outside the clinic.

Personal optimism (PO) may be especially relevant to this context. PO refers to generalized positive expectations regarding one’s own future, that is, the expectation that outcomes will be favorable irrespective of what or who brings that outcome about (Schweizer and Koch, 2001). Grounded in the behavioral self-regulation framework (Carver and Scheier, 1998; Carver et al., 2010), such expectancies represent not momentary positive affect but a relatively stable orientation that shapes how individuals appraise adversity, allocate effort, persist toward goals, and respond to uncertainty. Optimism so defined has been associated with better psychological and physical adjustment across health contexts (Rasmussen et al., 2009; Oh et al., 2022) and with adaptive responses during fertility treatment (Lancastle and Boivin, 2005).

A theoretical framework particularly relevant to prolonged treatment-related stress is Conservation of Resources (COR) theory (Hobfoll, 2001), which proposes that individuals strive to obtain, retain, and protect valued resources of various kinds. Resources do not operate in isolation; they can facilitate the acquisition or preservation of other resources, producing resource gain processes. Within COR theory, optimism has been explicitly identified as a key personal resource relevant to coping and adaptation (Hobfoll and Lilly, 1993). Accordingly, PO may shape how individuals appraise demands and mobilize available resources. In IVF, where outcomes are uncertain and treatment demands can extend over time, generalized positive expectancies may help individuals gain confidence in their capacity to cope. Nevertheless, PO need not be associated with ISE solely through an intrapsychic route. It may also operate through the way individuals organize and experience the competing demands of work, treatment, relationships, and personal life. We therefore propose the following hypothesis:

*H1*: Personal optimism (PO) will be positively associated with infertility self-efficacy (ISE) among women undergoing IVF treatment and their male partners.

Psychosocial vulnerability in this domain is neither confined to the treatment setting nor evenly distributed across patients. Population-level evidence indicates substantial unmet information and counseling needs in parenthood decision-making (Meyer et al., 2024). Relatedly, clinical depressiveness has been associated with distinct decision-making styles in the family-planning domain, including greater reliance on both rational and avoidant approaches (Lehnert et al., 2026). Although these studies do not examine ISE directly, they provide a broader psychosocial context for understanding why coping-related beliefs may vary among individuals facing fertility treatment and parenthood decisions. Evidence from within the treatment context points in the same direction: a systematic review of IVF studies found escapist coping strategies to be consistently associated with greater distress, whereas situation appraisals were among additional psychosocial factors associated with emotional adjustment in a smaller number of studies (Rockliff et al., 2014).

Work–life balance (WLB) refers to the extent to which individuals perceive that they are able to satisfactorily manage and integrate the demands of their work and personal lives (Greenhaus and Allen, 2011; Valcour, 2007). In this sense, WLB reflects the perceived compatibility between work and non-work roles and individuals’ satisfaction with how these domains are balanced. WLB may therefore provide a plausible contextual pathway through which optimism is linked to ISE. Fertility treatment often requires unpredictable appointments, medical procedures, emotional labor, decisions about disclosure, and concern about career consequences (Hadley et al., 2025; Wilkinson and Mumford, 2024). These demands can intensify time pressure and role conflict, making the preservation of WLB particularly consequential. Although fertility research has mainly treated occupational conditions as stressors associated with poorer adjustment or treatment outcomes (Matthiesen et al., 2011; Dehkordi et al., 2025; Turgeman-Lupo et al., 2025), positive integration across life domains may function as a resource that supports treatment-specific coping.

Existing research on optimism and research on occupational experiences during fertility treatment have largely proceeded separately. This division leaves unanswered whether a general positive orientation is associated with infertility-specific self-efficacy partly because optimistic individuals perceive greater manageability across their work and personal roles. It also obscures possible differences between partners. Infertility is experienced within a couple system, yet women and men may encounter different medical, occupational, and social demands.

The present study addresses these gaps by positioning PO as the focal personal resource and WLB as a potential intermediate correlate in the association between optimism and ISE. First, it examines whether generalized positive expectations are associated with a domain-specific coping belief partly through a potentially modifiable life condition. Second, it integrates the occupational sphere into fertility psychology by treating WLB as more than an outcome of stress: it may represent a pathway through which personal resources become relevant to coping during treatment. Third, it estimates the model separately for women and men while adjusting for the partner’s corresponding PO, WLB, and ISE scores, and then compares the two partner-specific indirect associations directly within a single dyadic model, thereby offering a couple-informed account of partner-differentiated resource configurations.

Perceived WLB may therefore represent a proximal contextual condition through which coping capacity is sustained during treatment. Fertility treatment places simultaneous demands on individuals’ time, energy, emotions, and occupational functioning (Hadley et al., 2025; Wilkinson and Mumford, 2024). When work and non-work roles are experienced as compatible, fewer resources may be lost to role conflict and depletion, leaving greater emotional and cognitive capacity for managing treatment procedures, uncertainty, setbacks, and difficult decisions. Such experiences of cross-domain manageability may strengthen individuals’ confidence that they can cope effectively with infertility and its treatment – that is, their ISE (Cousineau et al., 2006). We therefore hypothesize as follows:

*H2*: Perceived work–life balance (WLB) will be positively associated with ISE among women undergoing IVF treatment and their male partners.

Conservation of resources theory further suggests that resources operate in interconnected gain spirals, whereby an existing personal resource facilitates access to or preservation of contextual resources, which in turn strengthens individuals’ capacity to cope with demanding circumstances (Hobfoll, 2001). Although PO is generally regarded as a relatively stable personal resource, it may influence how individuals appraise demands and mobilize resources across life domains. Optimistic individuals are more likely to approach difficulties with persistence, engage in problem solving, seek support, and perceive competing demands as manageable (Carver et al., 2010; Hobfoll, 2001). During fertility treatment, these tendencies may facilitate the coordination of work responsibilities with medical appointments, treatment recovery, emotional demands, and couple life. This does not imply that optimism determines objective working conditions; rather, optimism may shape how individuals appraise, mobilize, and use the resources available within those conditions. Accordingly, higher PO is expected to be associated with greater perceived WLB. We therefore propose:

*H3:* PO will be positively associated with perceived WLB among women undergoing IVF treatment and their male partners.

Taken together, the reasoning underlying H2 and H3 suggests that these associations may form a sequential pathway. Accordingly, PO is expected to be associated with ISE not only directly, through favorable expectations and persistence, but also indirectly through its positive association with perceived WLB. We therefore propose:

*H4:* PO will show a positive indirect association with ISE through perceived WLB, such that higher PO will be associated with higher perceived WLB, which in turn will be associated with higher ISE.

Although PO, WLB, and ISE may all be relevant to coping for both partners, the pattern of associations among them may differ between women and men because they occupy different positions within the IVF process. Women typically experience the treatment more directly through ovarian stimulation, repeated monitoring, invasive procedures, and the physical and emotional consequences of treatment cycles (Ying et al., 2016). Their confidence in coping may therefore be shaped strongly by treatment-specific bodily and medical experiences, allowing PO and WLB to contribute relatively independently to ISE. By contrast, male partners generally experience fewer direct medical procedures, while coping with infertility through occupational demands, practical responsibilities, emotional support of their partners, and uncertainty regarding treatment outcomes (Ying et al., 2016; Turgeman-Lupo et al., 2025). Consequently, their generalized expectations may become connected with infertility-specific coping beliefs through their perceived capacity to integrate work and non-work demands. A COR perspective further suggests that resource-gain processes depend on individuals’ particular demands, roles, and opportunities to mobilize resources (Hobfoll, 2001). Because IVF-related burdens and occupational consequences may not be distributed identically within couples, the extent to which PO is associated with WLB, and WLB with ISE, may differ between partners. Previous dyadic research has similarly shown that occupational stressors can produce partner-differentiated patterns among couples undergoing IVF, emphasizing that apparently shared treatment circumstances do not necessarily operate through identical psychosocial pathways for women and men (Turgeman-Lupo et al., 2025). Nevertheless, the limited evidence regarding the direction of such differences does not justify predicting that the indirect association will be stronger for one partner. We therefore propose a nondirectional between-partner difference hypothesis:

*H5:* The magnitude of the indirect association between PO and ISE through perceived WLB will differ between women undergoing IVF treatment and their male partners.

## Materials and methods

### Study design

This study is a secondary analysis of cross-sectional data collected from women undergoing IVF treatment and their male partners at a tertiary hospital in Israel (*N* = 88; 44 couples). The dataset has been used in previous publications (Turgeman-Lupo et al., 2025; Bokek-Cohen et al., 2025), which examined occupational stress in relation to IVF outcomes and gender differences in multidimensional well-being. The present study addresses a distinct research question, and the hypotheses and findings reported here have not been published previously.

The study was approved by the Institutional Helsinki Committee for Medical Research in Humans, Meir Medical Center, Kfar Saba, Israel (approval no. 0276-21-MMC). All procedures were conducted in accordance with the Declaration of Helsinki. After providing written informed consent, both partners completed the self-report questionnaires separately. Completion time was approximately 30 min. To reduce potential interdependence bias during data collection, partners were explicitly instructed not to discuss their responses with one another while completing the questionnaires. Participation was voluntary, and no incentives were offered. Patients were not involved in the design of the study.

### Participants

Participants were recruited during their first visit to the IVF unit. Eligibility criteria included women aged 20–45 who were in a heterosexual relationship, were employed, and whose partners were also employed. Single women, women in same-sex partnerships, couples in which one partner was not employed, and women undergoing fertility preservation or surrogacy were excluded from the study. Patients who agreed to participate but whose partners declined were also excluded from the sample. A total of 44 couples met the eligibility criteria and agreed to participate (*N* = 88).

### Measures

All items were rated on a five-point Likert scale; scale scores were computed as item means, with higher scores indicating higher levels of the respective construct, and internal consistency was estimated using Cronbach’s α. PO was assessed using the four-item Personal Optimism subscale of the short POSO-E (Schweizer and Koch, 2001; Gavrilov-Jerković et al., 2014). Internal consistency of the original four-item score was low, particularly among men (*α* = 0.652 for women and 0.227 for men). The reverse-scored item “I worry about my future” showed poor psychometric fit with the remaining items and was therefore removed; this was a data-driven scoring decision. Its removal improved internal consistency (*α* = 0.718 for women and 0.527 for men), although internal consistency among men remained modest. Given this psychometric pattern, together with the possibility that future-related worry during IVF may reflect treatment-specific concern rather than generalized positive expectancies, the primary PO score was calculated as the mean of the remaining three items. WLB was assessed using a five-item scale adapted from Valcour (2007); internal consistency was α = 0.867 for women and 0.947 for men. ISE was assessed using the 16-item Infertility Self-Efficacy Scale developed and validated by Cousineau et al. (2006); internal consistency was α = 0.908 for women and 0.872 for men.

### Statistical analysis

A power analysis conducted in G*Power for five predictors, assuming *α* = 0.05, power = 0.90, and a large effect size of *f*^2^ = 0.50, indicated a minimum required sample of 39 couples. The same effect-size assumption (*f*^2^ = 0.50) used in the original sample-size calculation was retained. The sample of 44 couples exceeded the requirement for the five-predictor regression structure. This regression-based power calculation pertained to the five-predictor component regression and was not intended to establish statistical power for the indirect associations or for the between-partner contrast tested in H5. Hypotheses 1–4 were tested using IBM SPSS Statistics (version 29) and PROCESS (version 4.2; Hayes, 2022). Each partner-specific model adjusted for the other partner’s corresponding PO, WLB, and ISE scores, and indirect associations were estimated using 5,000 bootstrap resamples with 95% confidence intervals. To test Hypothesis 5, the two partner-specific indirect associations and their difference were estimated simultaneously in a distinguishable-dyad path model using the lavaan package (version 0.6-20; Rosseel, 2012) in R (version 4.4.2). Within the same dyadic model, the male-minus-female contrast between the partner-specific PO–WLB paths was also estimated directly. Percentile bootstrap confidence intervals were based on 5,000 couple-level resamples. Because H1–H4 were estimated in separate partner-specific PROCESS models, whereas the between-partner contrasts were estimated in the simultaneous dyadic model, the corresponding partner-specific coefficients may differ slightly across the two analyses. As a measurement sensitivity analysis, all focal analyses were repeated using the original four-item PO score; the results are reported in the Results section. Following conventional terminology (Hayes, 2022), the term “indirect effect” refers to the product of the a and b paths and is interpreted as a statistical indirect association rather than evidence of causal mediation.

## Results

### Participant characteristics

The analytic sample comprised 44 heterosexual couples (*N* = 88), all of whom met the study eligibility criterion that both partners were employed. Descriptive statistics for the sample characteristics, including women’s age and BMI, relationship duration, educational level and employment tenure for women and men, and clinical pregnancy outcome, are presented in Table 1. Means, standard deviations, and Pearson correlations among PO, WLB, and ISE for women and men are presented in Table 2.

**Table 1:** Descriptive statistics for sample characteristics.

Variable	Category	*R*	*M*	*SD*	*N* (%)
BMI	Female patients	19–41	25.50	4.92	44
Age (years)	Female patients	23–45	37.00	5.69	44
Relationship duration (years)	Couples	1–24	6.28	4.61	44
Education – female patients	High school	—	—	—	12 (27.3)
	Bachelor’s degree or student studying for a bachelor’s degree	—	—	—	23 (52.3)
	Master’s degree or higher	—	—	—	9 (20.5)
Education – male partners	High school	—	—	—	17 (38.6)
	Bachelor’s degree or student studying for a bachelor’s degree	—	—	—	21 (47.7)
	Master’s degree or higher	—	—	—	6 (13.6)
Employment tenure (years)	Female patients	1–16	5.52	4.24	44
	Male partners	1–30	6.09	5.39	44
Clinical pregnancy	Couples	—	—	—	20 (45.5)

R = observed range. BMI = body mass index.

**Table 2:** Means, standard deviations, and Pearson correlations among study variables.

Variable	*M*	*SD*	1	2	3	4	5
1. PO (women)	4.14	0.85	—				
2. WLB (women)	3.39	0.75	0.09	—			
3. ISE (women)	3.47	0.71	0.45**	0.56***	—		
4. PO (men)	4.39	0.69	0.00	0.25	0.22	—	
5. WLB (men)	3.65	0.84	0.04	−0.08	0.02	0.42**	—
6. ISE (men)	3.97	0.59	0.10	0.24	0.16	0.53***	0.51***

*N* = 44 couples (88 individuals). PO = Personal Optimism; WLB = Work-Life Balance; ISE = Infertility Self-Efficacy. *p < 0.05. **p < 0.01. ***p < 0.001.

### Regression diagnostics and effect sizes

VIF values ranged from 1.01 to 1.93, indicating no evidence of problematic multicollinearity. Histograms and normal P–P plots indicated no substantial departures from normality of the residuals. The models explained 15.4% of the variance in women’s WLB (95% CI for *R*^2^ [0.00, 0.29]; *f*^2^ = 0.18), 21.3% in men’s WLB (95% CI for *R*^2^ [0.00, 0.36]; *f*^2^ = 0.27), 48.7% in women’s ISE (95% CI for *R*^2^ [0.18, 0.60]; *f*^2^ = 0.95), and 42.6% in men’s ISE (95% CI for *R*^2^ [0.12, 0.55]; *f*^2^ = 0.74). The corresponding f^2^ values indicated medium overall model effects for the two WLB models and large overall model effects for the two ISE models.

### Hypothesis testing

Hypothesis 1 proposed that PO would be positively associated with ISE among both partners. This hypothesis was supported. Among women, PO was positively associated with ISE after adjustment for WLB and the male partner’s corresponding PO, WLB, and ISE scores, *B* = 0.339, *SE* = 0.098, *t* = 3.45, *p* = 0.001, 95% CI [0.140, 0.537]. Among men, PO was likewise positively associated with ISE after adjustment for WLB and the female partner’s corresponding scores, *B* = 0.291, *SE* = 0.123, *t* = 2.37, *p* = 0.023, 95% CI [0.043, 0.539].

Hypothesis 2 proposed that perceived WLB would be positively associated with ISE among both partners. This hypothesis was also supported. Perceived WLB was positively associated with ISE among women, *B* = 0.490, *SE* = 0.119, *t* = 4.12, *p* < 0.001, 95% CI [0.249, 0.730], and among men, *B* = 0.270, *SE* = 0.097, *t* = 2.77, *p* = 0.009, 95% CI [0.073, 0.467]. The full ISE equations explained 48.7% of the variance among women, *R*^2^ = 0.487, *F*(5, 38) = 7.20, *p* < 0.001, and 42.6% among men, *R*^2^ = 0.426, *F*(5, 38) = 5.65, *p* < 0.001.

Hypothesis 3 proposed that PO would be positively associated with perceived WLB among both partners. Among women, the association was not statistically significant, *B* = 0.070, *SE* = 0.132, *t* = 0.53, *p* = 0.601, 95% CI [−0.197, 0.336]; the WLB equation was not significant overall, *R*^2^ = 0.154, *F*(4, 39) = 1.77, *p* = 0.154. Among men, PO was positively associated with perceived WLB, *B* = 0.566, *SE* = 0.180, *t* = 3.14, *p* = 0.003, 95% CI [0.202, 0.930], and the WLB equation explained 21.3% of the variance, *R*^2^ = 0.213, *F*(4, 39) = 2.64, *p* = 0.048. Thus, H3 was supported among men but not among women. To determine whether the partner-specific PO–WLB associations differed directly, the two paths were compared within the simultaneous distinguishable-dyad model. In this model, PO was positively associated with WLB among men, *B* = 0.507, *SE* = 0.197, *p* = 0.010, 95% bootstrap CI [0.102, 0.871], but not among women, *B* = 0.080, *SE* = 0.147, *p* = 0.588, 95% bootstrap CI [−0.208, 0.391]. The male-minus-female contrast was statistically different from zero, Δ*B* = 0.427, 95% percentile bootstrap CI [0.002, 0.821]. Because the lower confidence bound was very close to zero, this difference should be interpreted cautiously.

Hypothesis 4 proposed a positive indirect association between PO and ISE through perceived WLB. Among women, the indirect effect was not statistically different from zero, indirect effect = 0.034, *BootSE* = 0.072, 95% bootstrap CI [−0.115, 0.181]. The total association between PO and ISE was significant, *B* = 0.373, *SE* = 0.116, *t* = 3.21, *p* = 0.003, 95% CI [0.138, 0.607]. Among men, the indirect effect was statistically different from zero, indirect effect = 0.153, *BootSE* = 0.079, 95% bootstrap CI [0.022, 0.330]. The total association between PO and ISE was also significant, *B* = 0.444, *SE* = 0.118, *t* = 3.75, *p* < 0.001, 95% CI [0.204, 0.683]. Accordingly, H4 was supported among men but not among women. The partner-specific path estimates are presented in Tables 3, 4 and illustrated in Figures 1, 2.

**Table 3:** Indirect-effect analysis of personal optimism, work-life balance, and infertility self-efficacy among women undergoing IVF.

Path/effect	*B*	*SE*	*t*	*p*	95% CI
PO → ISE (direct; H1)	0.339	0.098	3.45	0.001	[0.140, 0.537]
WLB → ISE (H2)	0.490	0.119	4.12	<0.001	[0.249, 0.730]
PO → WLB (H3)	0.070	0.132	0.53	0.601	[−0.197, 0.336]
PO → ISE (total)	0.373	0.116	3.21	0.003	[0.138, 0.607]
PO → WLB → ISE (indirect; H4)	0.034	0.072	—	—	[−0.115, 0.181]

B = unstandardized coefficient; PO = Personal Optimism; WLB = Work-Life Balance; ISE = Infertility Self-Efficacy. Models adjusted for the male partner’s corresponding PO, WLB, and ISE scores. For the indirect effect, the SE and 95% CI are based on 5,000 bootstrap resamples; the 95% CI is the percentile bootstrap confidence interval. WLB equation: *R*^2^ = 0.154, *F*(4, 39) = 1.77, *p* = 0.154. ISE equation: *R*^2^ = 0.487, *F*(5, 38) = 7.20, *p* < 0.001.

**Table 4:** Indirect-effect analysis of personal optimism, work-life balance, and infertility self-efficacy among male partners.

Path/effect	*B*	*SE*	*t*	*p*	95% CI
PO → ISE (direct; H1)	0.291	0.123	2.37	0.023	[0.043, 0.539]
WLB → ISE (H2)	0.270	0.097	2.77	0.009	[0.073, 0.467]
PO → WLB (H3)	0.566	0.180	3.14	0.003	[0.202, 0.930]
PO → ISE (total)	0.444	0.118	3.75	<0.001	[0.204, 0.683]
PO → WLB → ISE (indirect; H4)	0.153	0.079	—	—	[0.022, 0.330]

B = unstandardized coefficient; PO = Personal Optimism; WLB = Work-Life Balance; ISE = Infertility Self-Efficacy. Models adjusted for the female partner’s corresponding PO, WLB, and ISE scores. For the indirect effect, the SE and 95% CI are based on 5,000 bootstrap resamples; the 95% CI is the percentile bootstrap confidence interval. WLB equation: *R*^2^ = 0.213, *F*(4, 39) = 2.64, *p* = 0.048. ISE equation: *R*^2^ = 0.426, *F*(5, 38) = 5.65, *p* < 0.001.

**Figure 1:**
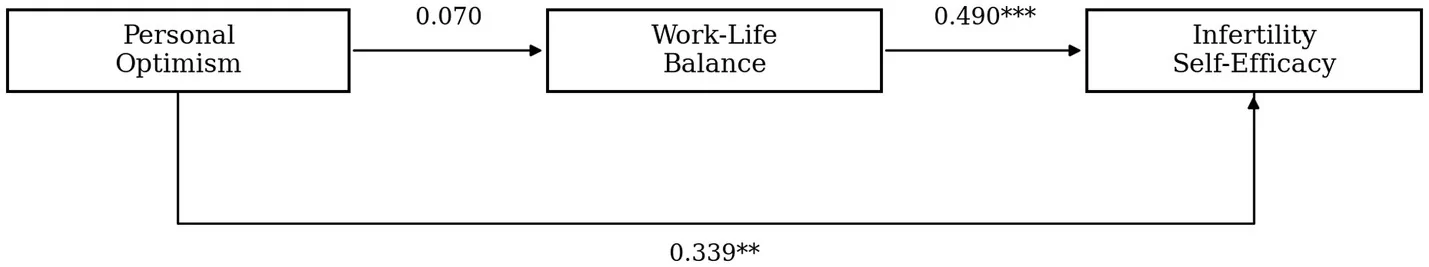
Indirect-effect model for women undergoing IVF. Unstandardized coefficients are shown on the paths. Indirect effect = 0.034, 95% percentile bootstrap CI [−0.115, 0.181], based on 5,000 resamples. *p < 0.05. **p < 0.01. ***p < 0.001.

**Figure 2:**
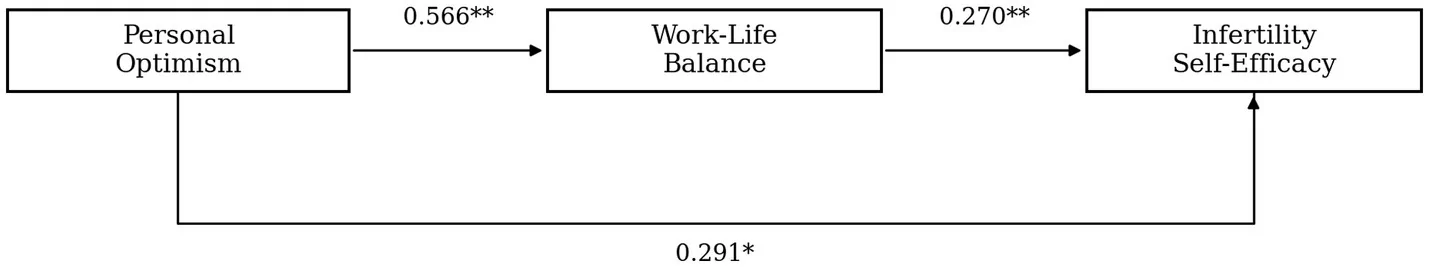
Indirect-effect model for male partners. Unstandardized coefficients are shown on the paths. Indirect effect = 0.153, 95% percentile bootstrap CI [0.022, 0.330], based on 5,000 resamples. *p < 0.05. **p < 0.01. ***p < 0.001.

Hypothesis 5 proposed that the magnitude of the indirect association would differ between women and men. In the simultaneous distinguishable-dyad model, the indirect association was not statistically different from zero among women, *B* = 0.038, 95% bootstrap CI [−0.115, 0.173], whereas it was statistically different from zero among men, *B* = 0.137, 95% bootstrap CI [0.013, 0.306]. However, the male-minus-female contrast was not statistically different from zero, Δ*B* = 0.099, 95% bootstrap CI [−0.091, 0.314]. Thus, H5 was not supported.

### Sensitivity analysis

All focal analyses were repeated using the original four-item PO score. The partner-specific indirect-association pattern was unchanged: the indirect association remained nonsignificant among women and significant among men, while the direct comparison between the two indirect associations remained nonsignificant. The direct association between PO and ISE among men was attenuated and narrowly missed conventional significance (*p* = 0.053). Thus, the central indirect-association findings were robust to retaining the original four-item PO measure.

## Discussion

Taken together, the findings indicate that PO and WLB were each associated with ISE in both women undergoing IVF and their male partners. A direct comparison within the simultaneous dyadic model indicated that the PO–WLB association was stronger among men than among women; however, because the lower confidence bound for this contrast was very close to zero, this difference should be interpreted cautiously. These associations were estimated while adjusting for the other partner’s corresponding PO, WLB, and ISE scores. This couple-informed analytic approach accounts for some partner-level covariation and allows the focal associations to be interpreted as partner-specific associations within the couple context. In this way, the findings underscore the methodological and conceptual value of considering both partners when examining psychosocial functioning in fertility treatment.

Importantly, the observed configuration of these associations differed between women and men. Among men, higher PO was associated with higher WLB, and WLB was in turn associated with higher ISE, yielding an indirect PO–WLB–ISE association. Among women, PO and WLB were each independently associated with ISE, whereas PO was not significantly associated with WLB. These findings suggest that PO and WLB are relevant to ISE in both partners, while their observed interrelations differed. Importantly, the statistically significant between-partner contrast concerned the PO–WLB path specifically. The direct comparison of the partner-specific indirect effects remained nonsignificant; therefore, the findings support a partner difference in the PO–WLB association but do not establish a between-partner difference in the overall indirect association.

From a broader psychosocial perspective, the present findings shift attention from ISE solely as an outcome of adjustment to broader factors associated with this treatment-specific coping belief. ISE reflects perceived self-efficacy for coping with infertility treatment (Cousineau et al., 2006), whereas PO reflects generalized positive expectations regarding one’s own future (Schweizer and Koch, 2001; Gavrilov-Jerković et al., 2014). Their positive association in both partners suggests that broad future-oriented expectancies may be relevant to more treatment-specific coping beliefs. A meta-analytic review linked optimism to better physical health outcomes (Rasmussen et al., 2009), and longitudinal work found that baseline optimism predicted later physical health and psychological well-being (Oh et al., 2022). Within fertility treatment, Lancastle and Boivin (2005) identified dispositional optimism as one component of a broader latent psychological factor that predicted biological response to treatment, whereas Abramov et al. (2022) found that unrealistic optimism regarding IVF success was associated with better psychological adjustment. The present study extends this literature by showing that PO, operationalized as generalized positive expectations regarding one’s own future, was associated with infertility-specific self-efficacy in both partners.

The present findings also broaden understanding of ISE by highlighting the relevance of the occupational domain. Research on the work–fertility interface has documented work–life challenges associated with fertility treatment, including treatment appointments and work absences, disclosure dilemmas, and concerns about workplace consequences and support (Wilkinson and Mumford, 2024; Hadley et al., 2025). In the present study, WLB was positively associated with ISE in both partners, suggesting that greater satisfaction with balancing work and personal-life demands is meaningfully related to confidence in coping with infertility treatment. This aligns with a previous study that reported associations between women’s job-related stress and emotional exhaustion and IVF pregnancy outcome (Turgeman-Lupo et al., 2025). More broadly, the findings indicate that coping-related beliefs during fertility treatment are related not only to intrapersonal psychological resources but also to the wider life context in which treatment is managed.

The joint relevance of PO and WLB can also be understood within Conservation of Resources (COR) theory. As presented above, COR theory holds that individuals strive to obtain, retain, foster, and protect valued resources and that stress is especially likely when resources are threatened or lost (Hobfoll, 2001; Hobfoll et al., 2018). Within this perspective, PO represents a personal resource, consistent with Hobfoll and Lilly’s (1993) explicit identification of optimism as a key resource relevant to coping and adaptation, whereas WLB, as operationalized here, reflects satisfaction with how work and personal-life demands are balanced (Valcour, 2007). Their independent associations with ISE in both partners are consistent with the idea that coping confidence is embedded in a broader resource context rather than residing solely within the individual. Among men, the additional association between PO and WLB suggests that personal and contextual appraisals may be more closely linked, such that positive future expectations coincide with a stronger sense of balance across life domains. At the level of the partner-specific analyses, however, the pattern corresponding to the proposed resource-gain sequence was observed among men but not among women: among men, PO was associated with WLB, which was in turn associated with ISE, whereas among women, PO and WLB were each associated with ISE without being significantly associated with one another. This pattern suggests that the application of COR gain-spiral logic to the proposed PO–WLB–ISE sequence may depend on the conditions under which contextual resources are experienced and maintained.

One possible explanation is that the WLB of the partner undergoing the medical procedures is especially sensitive to treatment-related work disruptions, including repeated appointments, absences, disclosure decisions, and the availability of workplace support, thereby making WLB less closely tied to generalized future expectations. This interpretation is consistent with evidence that women undergo the more intensive medical procedures of IVF and with work-fertility research documenting substantial employment-related difficulties during treatment (Ying et al., 2016; Wilkinson and Mumford, 2024; Hadley et al., 2025). Given the modest sample size, sampling variability is also a plausible alternative explanation for the nonsignificant PO–WLB association and indirect association among women, and these findings should therefore be examined in larger dyadic samples.

The partner-specific pattern should also be considered in light of the broader couple-based infertility literature. Peterson et al. (2006) documented gender differences in the relative use of coping strategies among couples referred for IVF, and a systematic review by Ying et al. (2016) found that emotional reactions to IVF differed between women and men across treatment stages. More recently, Khorasani et al. (2025) found in a systematic review and meta-analysis that women reported higher anxiety, depression, and stress and lower infertility self-efficacy than men. The present findings add to this literature by suggesting that PO, WLB, and ISE may form somewhat different configurations across partners while remaining relevant to both. Importantly, the absence of a significant direct difference between the indirect effects means that these patterns should not be interpreted as evidence of categorically different processes in women and men. Rather, they point to a potentially meaningful partner-specific configuration.

From a psychosocial support perspective, these findings are relevant to routine fertility care, where ESHRE guidance recommends attention to patients’ psychosocial needs across the treatment pathway, including relational needs that encompass work (Gameiro et al., 2015). Because ISE was associated with both PO and WLB, attention to patients’ coping confidence, broader psychological resources, and work–life circumstances may help fertility teams develop a more complete picture of psychosocial needs. In particular, brief discussion of work scheduling, treatment-related absences, disclosure concerns, and available workplace support may help identify practical pressures that accompany treatment (Wilkinson and Mumford, 2024; Hadley et al., 2025). Prior intervention research has shown that infertility-related self-efficacy can be increased through fertility-supporting health training and positive psychotherapy-based training (Altiparmak and Derya, 2018; Yanık and Kavak Budak, 2024). The present findings therefore highlight the potential value of broadening psychosocial care beyond intrapersonal factors to include patients’ wider life circumstances, including their work environments. Looking beyond the treatment period itself, coping-related beliefs formed during fertility treatment may also be relevant to longer-term adjustment to parenthood. In a large German panel study, 11% of participants reported that they would probably or definitely not choose to have children again, and regretting parenthood was associated with more pronounced depressive symptoms, greater perceived psychosocial costs of raising children, younger parental age, and lower socioeconomic status, but not with perceived benefits of parenthood (Erbe et al., 2025). That study did not assess ISE, and the present cross-sectional data cannot speak to post-treatment outcomes. It nonetheless indicates that the psychosocial trajectory of individuals pursuing parenthood extends well beyond conception, and it suggests that strengthening coping resources during treatment may be worth examining in relation to longer-term parental adjustment in future longitudinal research.

Several limitations of the present study should be acknowledged. First, the cross-sectional design precludes causal conclusions regarding the directionality of the associations observed. This concern applies with particular force to the PO–WLB path. The focal model specified PO as the antecedent of perceived WLB based on the theoretical rationale developed above, but the data cannot adjudicate between this ordering and its reverse. Although PO is conceptualized as a relatively stable, trait-like orientation (Schweizer and Koch, 2001; Carver et al., 2010), relatively stable characteristics are not insulated from contextual influence, and sustained experiences of manageable or unmanageable work and non-work demands may equally shape generalized positive expectations. Reciprocal processes are likewise plausible within COR theory, which describes resources as mutually reinforcing over time (Hobfoll, 2001). The PO–WLB association reported here should therefore be read as compatible with either ordering rather than as evidence that optimism shapes perceived balance. Longitudinal studies are needed to clarify temporal ordering, including the possibility of reciprocal effects, and to examine whether changes in PO and WLB predict subsequent changes in ISE over the course of treatment. Second, the relatively small sample size and recruitment from a single clinical site may limit generalizability to broader populations of fertility patients. The modest sample size may also limit the precision of the estimated effects, particularly the direct comparison of the partner-specific indirect associations. The sample was also restricted to employed heterosexual couples, and the medically treated partner was always the woman; accordingly, gender differences cannot be separated fully from differences in treatment role. Third, all measures were based on self-report, which may introduce shared method variance. Fourth, the internal consistency of the PO measure was low among men. Although removal of the reverse-scored item improved Cronbach’s α from 0.227 to 0.527, internal consistency remained modest. Because measurement unreliability may attenuate observed associations and reduce their precision, this limitation is particularly relevant to the male partner-specific analysis, in which the significant indirect association was observed. At the same time, the sensitivity analysis using the original four-item score reproduced the central indirect-association pattern, indicating that this pattern was not dependent on the scoring decision. Nevertheless, the sensitivity analysis does not eliminate the underlying reliability limitation, which should be considered when interpreting the magnitude and precision of associations involving male PO. Future studies would benefit from more detailed occupational information, multi-method assessments, larger and more diverse dyadic samples, and repeated assessments across treatment stages. Finally, although both partners’ scores were included in all analyses, the present study was not designed as a full dyadic test of interpersonal processes. The partner’s PO, WLB, and ISE scores were entered as statistical adjustments, in order to isolate partner-specific associations within the couple context, rather than as theorized actor–partner effects. Accordingly, we did not formulate hypotheses about, and do not interpret, crossover pathways in which one partner’s resources are associated with the other partner’s coping beliefs. Future dyadic studies with larger samples could formulate and test explicit hypotheses concerning actor and partner pathways and interpret these interpersonal effects directly.

## Conclusion

In the present study, personal optimism and work–life balance were associated with infertility self-efficacy among women undergoing IVF and their male partners, with partner-specific patterns observed. Among men, PO showed an indirect association with ISE through WLB, whereas among women, PO and WLB were independently associated with ISE. The direct comparison between the partner-specific indirect effects was not significant, indicating that the findings are better understood as a difference in observed configuration than as evidence of a confirmed difference in the indirect effect between partners. Overall, the results highlight the potential relevance of both psychological and work–life factors for understanding psychosocial adaptation in IVF and extend current work by positioning personal optimism and work–life balance as complementary correlates of infertility-specific coping confidence within the couple context. These findings may inform the development of psychosocial support for couples during this stressful period in their lives. Given the cross-sectional design and modest sample size, these findings should be confirmed in larger longitudinal studies, and intervention research is needed before the clinical value of targeting PO or WLB can be established.

## Data Availability

The dataset analyzed in this study contains sensitive clinical and psychosocial information collected from couples undergoing fertility treatment. Due to ethical and privacy considerations and restrictions imposed by the institutional ethics committee, the data cannot be made publicly available. De-identified data may be available from the corresponding author upon reasonable request and subject to ethics approval. Requests to access these datasets should be directed to kerentu2@mta.ac.il.
